# Muscle-Organ Crosstalk: Focus on Immunometabolism

**DOI:** 10.3389/fphys.2020.567881

**Published:** 2020-09-09

**Authors:** Marie Lund Bay, Bente Klarlund Pedersen

**Affiliations:** Centre of Inflammation and Metabolism/Centre for Physical Activity Research (CIM/CFAS), Rigshospitalet, University of Copenhagen, Copenhagen, Denmark

**Keywords:** metabolism, cytokines, exercise, physical activity, diabetes, cancer

## Abstract

Skeletal muscle secretes several hundred myokines that facilitate communication from muscle to other organs, such as, adipose tissue, pancreas, liver, gut, and brain. The biological roles of myokines include effects on e.g., memory and learning, as well as glucose and lipid metabolism. The present minireview focuses on recent developments showing that exercise-induced myokines are involved in immunometabolism of importance for the control of e.g., tumor growth and chronic inflammation. In this review, immunometabolism is discussed as the non-immune related pathologies leading to an immune response and some degree of inflammation, which promotes metabolic abnormalities.

## Introduction

The term “immunometabolism” was introduced as the interplay between metabolic and immunologic processes (Mathis and Shoelson, 2011). Immunometabolism refers to two concepts. One is how leukocyte and lymphocyte function is regulated by their internal metabolism. The other is how pathologies considered to be non-immune – such as obesity – result in an activation of the immune system, which promotes metabolic abnormalities increasing the risk of type 2 diabetes, cardiovascular diseases and cancer (Mathis and Shoelson, 2011). In this review, the main focus is on the latter understanding of the immunometabolism concept, and on how muscle activation through exercise can counteract some of the inflammatory processes related to these diseases.

Myokines are involved in mediating the multiple physiological, metabolic and immunological effects of physical activity (Pedersen et al., 2003a; Pedersen and Febbraio, 2012).

A single bout of exercise provokes an increase in systemic levels of IL-6 (Pedersen, 2013). In relation to exercise, IL-6 is released as a myokine from muscle into the circulation, and IL-6 plasma levels increase exponentially with exercise duration. Recent findings consolidate the pleiotropic nature of IL-6 and demonstrate a physiological role of this myokine in regulating clinically relevant parameters related to energy homeostasis and immune cell regulation in cancer (Severinsen and Pedersen, 2020).

**FIGURE 1:**
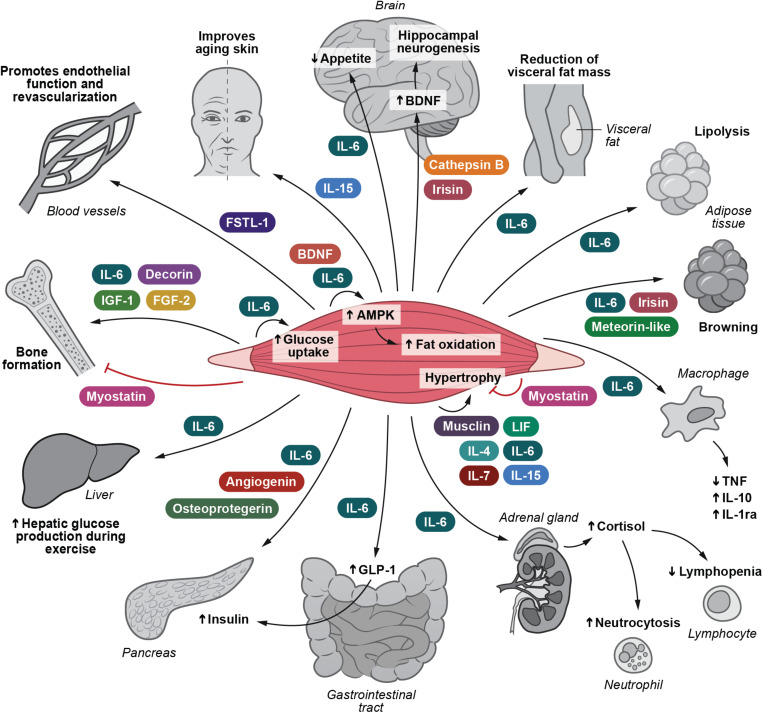
I Irisin and Cathepsin B enhance BDNF production and thereby hippocampal neurogenesis. IL-6 inhibits appetite and stimulates lipolysis. IL-6 also plays a role in decreasing the amount of visceral fat. IL-6, irisin and meteorin-like are involved in turning white adipose tissue into a brown phenotype. IL-15 retards skin aging. IL-6, decorin, FGF-2 and IGF-1 positively influence bone formation. Myostatin negatively influence bone formation. Musclin, LIF, IL-4, IL-6, IL-7, and IL-15 are involved in mediating muscle hypertrophy, whereas myostatin obstructs muscle hypertrophy. IL-6 and BDNF stimulate AMPK activation and hence fat oxidation. IL-6 e stimulates glucose uptake and hepatic glucose output during exercise. IL-6 induces the expression of GLP-1 by the L cells of the intestine leading to enhanced insulin secretion. IL-6 exerts anti-inflammatory effects by inhibiting TNF production and by stimulating IL-1ra and IL-10 production. IL-6 enhances cortisol production, leading to neutrocytosis and lymphopenia. FSTL-1 has beneficial effects on endothelial function and revascularization of atherosclerotic blood vessels. Osteoprotegerin, angiogenin, and IL-6 possess beta-cell protective actions against inflammatory cytokines. AMPK, 5’-AMP-activated protein kinase; BDNF, brain-derived neurotrophic factor; FGF-2, fibroblast growth factor 2; FGF-21, fibroblast growth factor 21; FSTL-1, follistatin-related protein 1; GLP-1, glucagon-like peptide 1; IGF-1, insulin-like growth factor I; IL-1ra, IL-1 receptor antagonist; LIF, leukemia inhibitory factor; TGF-β, transforming growth factor β; TNF, tumor necrosis factor. Adapted with permission from Severinsen and Pedersen (2020).

Following the identification of muscle-derived IL-6, it has become evident that skeletal muscle cells are able to secrete more than 650 myokines (Khan and Ghafoor, 2019). The role of myokines has previously been reviewed (Pedersen et al., 2007; Pedersen, 2009, 2011, 2019; Walsh, 2009; Brandt and Pedersen, 2010; Arnold et al., 2011; Hamrick, 2011; Trayhurn et al., 2011; Pedersen and Febbraio, 2012; Raschke and Eckel, 2013; Pal et al., 2014; Ahima and Park, 2015; Benatti and Pedersen, 2015; Indrakusuma et al., 2015; Schnyder and Handschin, 2015; Whitham and Febbraio, 2016; Hoffmann and Weigert, 2017; Rodriguez et al., 2017; Ruiz-Casado et al., 2017; Diaz et al., 2018; Fiuza-Luces et al., 2018; Coelho-Junior et al., 2019; Das et al., 2019; Eckel, 2019; Garneau and Aguer, 2019; Graf and Ferrari, 2019; Lee and Jun, 2019). Until now the biological function has been described for only approximately 5% of all known myokines. Nonetheless, the identification of the myokinome has provided a new paradigm and a conceptual basis for understanding by which mechanisms muscles communicate with other organs. Several of these myokines relate to immune function and inflammation. Low-grade inflammation is associated with several types of obesity-related diseases such as diabetes, cardiovascular disease, cirrhosis, and cancer. We suggest that control of this pathology-related inflammation can in part be ascribed by the release of immunogenic myokines. These are highlighted in Figure 1.

## Muscle-Immune-Inflammation Crosstalk

Muscle has impact on lymphocyte and neutrophil trafficking and inflammation (Duggal et al., 2019). During exercise, neutrophils as well as natural killer (NK) cells and other lymphocytes enter the blood. Exercise of high intensity and long duration leads to a decline in lymphocyte number, while the concentration of neutrophils increase (McCarthy and Dale, 1988; Pedersen and Hoffman-Goetz, 2000) via mechanisms that include adrenaline and cortisol. IL-6 has been shown to be involved in mediating the increase in cortisol (Steensberg et al., 2003).

Lack of exercise and obesity are accompanied by low level chronic inflammation (Pedersen et al., 2003b; Petersen and Pedersen, 2005; Pedersen, 2006; Pedersen, 2006, 2017; Benatti and Pedersen, 2015; Knudsen and Pedersen, 2015; Karstoft and Pedersen, 2016). The anti-inflammatory effects of exercise training are induced with each single bout of exercise as well as via training adaptation leading to a decrease in the amount of abdominal fat.

IL-6 increases with exercise and promotes the occurring of two cytokines with anti-inflammatory effects (Steensberg et al., 2003). IL-1 receptor antagonist (IL-1ra) blocks IL-1β signaling (Dinarello, 1994) and IL-10 prevents TNF-α production (Opp et al., 1995). A study in healthy humans showed that a bout of exercise or administration of IL-6 before infusion of endotoxin abolished the increase in plasma levels of TNF-α that was seen in a control situation (Starkie et al., 2003). It was concluded that a single bout of exercise mediates an anti-inflammatory signal, which is likely to be partly mediated by IL-6 (Pedersen, 2017).

Exercise can also induce anti-inflammatory effects via a reduction in abdominal fat (Rosenkilde et al., 2016). Abdominal adiposity, reflecting a high amount of visceral fat, is associated with cardiovascular disease, type 2 diabetes, dementia and all-cause mortality (Pedersen, 2009). Accumulation of visceral fat represents an important source of origin of chronic systemic inflammation, as it has been shown to be more inflamed than subcutaneous fat, constituting an important source of inflammatory markers (Yudkin, 2007).

Physical inactivity leads to an increased amount of visceral fat and consequently an environment of inflammation, which provokes a network of chronic diseases (Benatti and Pedersen, 2015).

Recent evidence exists that exercise training decreases the amount of visceral and cardiac fat mass (Christensen et al., 2019a,b; Wedell-Neergaard et al., 2019) mediated by muscle-derived IL-6 (Wedell-Neergaard et al., 2019) as described below.

## Muscle-Adipose Crosstalk

Exercise-induced IL-6 has significant effects on fat metabolism (Pedersen, 2013, 2018). *In vivo* studies in humans show that rhIL-6 enhances lipolysis and fat oxidation (van Hall et al., 2003; Petersen et al., 2005). Epidemiological studies demonstrate that an association exists between abdominal adiposity and low fitness (Wedell-Neergaard et al., 2018a,b). Intervention studies show that reduced number of daily steps provoke accumulation of visceral adipose tissue (Olsen et al., 2008; Benatti and Pedersen, 2015), whereas exercise training reduced abdominal adiposity (Ross et al., 2000; Nordby et al., 2012). In a recent study, abdominally obese humans were randomized to tocilizumab (IL-6 receptor antibody) or placebo during an intervention of 12-weeks with either aerobic exercise or no exercise (Christensen J. F. et al., 2018; Wedell-Neergaard et al., 2019). While exercise training led to a reduction in visceral adipose tissue mass, this effect was completely abolished by IL-6 receptor blockade (Wedell-Neergaard et al., 2019).

It has also been hypothesized that exercise may induce browning of white adipose tissue (Rodriguez et al., 2017; Eckel, 2019; Townsend and Wright, 2019). Myokines with browning properties include irisin (Bostrom et al., 2012), meteorin-like (Rao et al., 2014), and IL-6 (Knudsen et al., 2014). The finding that exercise-induced myokines may induce browning of white adipose tissue has been demonstrated in rodent models, but not consistently so in humans (Norheim et al., 2014; Vosselman et al., 2015; Severinsen et al., 2020).

## Muscle-Cancer Crosstalk

Metabolic syndrome has been tied to risk of several types of cancer (Esposito et al., 2012). The vast amount of epidemiological studies demonstrate that exercise training decreases the risk of cancer and contributes to control disease progression. Exercise has also beneficial impact on anti-cancer therapy and improves physical and mental health in general. Being physically active reduces the risk of approximately 13 different cancer types (Moore et al., 2016; Pedersen et al., 2016; Christensen R. H. et al., 2018; Hojman et al., 2018). Exercise training after a diagnosis of breast cancer, prostate cancer and colorectal cancer are associated with an increased survival rate (Pedersen, 2018).

Given that cancer is associated with low level chronic inflammation, which may contribute to tumor progression, it is possible that the ability of physical training to create an anti-inflammatory environment, may facilitate exercise-induced protection on cancer growth (Hojman et al., 2018).

Pernille Hojman studied exercise effects on tumor growth in rodent models (Pedersen et al., 2016). She established a B16F10 melanoma model and exposed tumor-bearing mice to wheel running or control. It appeared that exercising mice had a significant decrease in tumor mass and incidence.

Myokines are involved in mediating the effect of exercise on tumor growth. When breast cancer cells were treated with irisin, they were more likely to undergo apoptosis (Hojman et al., 2018).

The myokine oncostatin M (Pedersen et al., 2016) was shown to inhibit breast cancer cell proliferation. The myokine, secreted protein acidic and rich in cysteine (SPARC) was shown to reduce tumor in the colon of exercising mice (Aoi et al., 2013).

Exercise also induces acute increases in epinephrine and norepinephrine, which are involved in recruiting NK cells in humans during exercise. Breast cancer cells exposed to serum collected after a single bout of acute exercise and thereafter injected into mice, led to a reduction of tumor formation (Dethlefsen et al., 2017). This effect was, however, completely blunted when we blocked β-adrenergic signaling, the pathway through which epinephrine and norepinephrine work (Dethlefsen et al., 2017).

These findings suggested that epinephrine and norepinephrine play a key role in the cancer-inhibiting effects of exercise. To this end, catecholamine release has been linked to the best-characterized myokine, IL-6, which increases exponentially during exercise in humans. Muscle cells from rats have been shown to release IL-6 upon stimulation with epinephrine (Frost et al., 2004), and injection of a high dose of IL-6 in human subjects resulted in increased epinephrine levels (van Hall et al., 2003).

**FIGURE 2:**
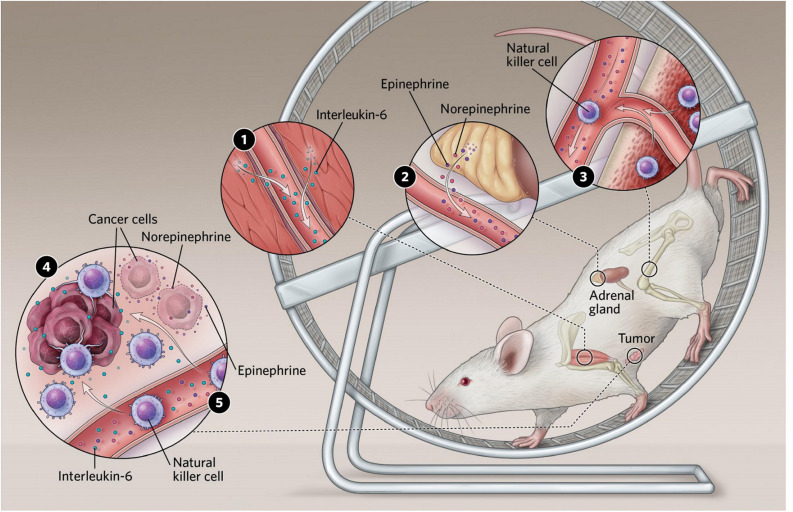
**(1)** Exercising muscles release multiple compounds known as myokines. Several of these have been shown to affect cancer cell proliferation in culture, and some, including interleukin-6, slow tumor growth in mice. **(2)** Exercise stimulates an increase in levels of the stress hormones epinephrine and norepinephrine, which can both act directly on tumors and stimulate immune cells to enter the bloodstream. **(3)** Epinephrine also stimulates natural killer cells to enter circulation. **(4)** In mice, interleukin-6 appears to direct natural killer cells to home in on tumors. Reprinted with permission from Pedersen (2020). Illustrator: Scott Leighton. **(5)** Epinephrine and norepinephrine along with some myokines can inhibit tumor growth.

In the cancer-setting, Pernille Hojman and her team found that the inhibitory effects of exercise on tumor growth were mediated via a direct regulation of natural killer (NK) cells, where these were mobilized to the circulation and redistributed to the tumor tissue by a mechanism involving both epinephrine and IL-6. Blocking IL-6 signaling during exercise abolished the exercise-induced inhibition of tumor growth, suggesting that IL-6 plays a role in mediating anti-cancer effects (Aoi et al., 2013; Hojman et al., 2011; Hojman et al., 2018; Lucia and Ramirez, 2016; Manole et al., 2018; Figure 2).

In addition to the crucial increase of tumor-infiltrating NK cells with exercise, microarray analyses of the tumors revealed that 52% of the upregulated gene ontology pathways were linked to immunological and inflammatory responses, and qPCR analyses showed increased tumoral expression of several cytokines (Pedersen et al., 2016). Amongst these upregulated cytokines were Interferon-γ, which has been reported to stimulate immunoregulatory molecules on a wide selection of both healthy and diseased cells (Sun et al., 2018), and IL-15 known to stimulate activation and cytotoxicity of both NK cells and T cells (Guo et al., 2017). These clear associations between exercise and the immunogenic profiles of tumors makes it higly relevant to study the possible benefits of combining exercise with immunotherapy. These could either be checkpoint inhibitors or immune-stimulatory treatments.

## Muscle-Cardiac Tissue Crosstalk

The inflamed arterial wall is a hallmark in the development of cardiovascular disease. Given that each bout of exercise induces anti-inflammatory effects, mediated by IL-6, it is likely that transient increases in this myokine contributes to the protection against atherosclerotic disease.

Another myokine of importance for cardiac disease is follistatin-like 1 (FSTL1), which is expressed by skeletal as well as cardiac muscle cells (Shimano et al., 2012). FSTL1 promotes the function of endothelial cells and is involved in revascularization (Oshima et al., 2008; Ouchi et al., 2008), although its role in humans need to identified.

## Muscle-Liver Crosstalk

Exercise stimulates an augmented production of glucose from the liver (Wasserman et al., 1991). In 1961, Goldstein (1961) proposed that contracting muscle produced an exercise factor that could stimulate hepatic glucose output. Evidence exists that IL-6 plays a role in hepatic glucose output. This was the conclusion from a study in which young healthy males did 2 h of cycle ergometer exercise on 3 different days at: (1) a high intensity; (2) a low intensity; and (3) a low intensity + infusion of IL-6 at a concentration to mimic the systemic increase in IL-6 during exercise of high intensity. The results from this human experiment demonstrated that exercise-induced IL-6 is involved in triggering hepatic glucose output during exercise (Febbraio et al., 2004).

### Muscle-Beta-Cell

Studying human primary muscle cell cultures established from triceps brachii, soleus and quadriceps identified two myokines, angiogenin and osteoprotegerin, which were shown to be triceps specific myokines, mediating anti-inflammatory actions and protecting beta-cell survival (Rutti et al., 2018). Moreover, it has been shown that IL-6 positively regulates β-cell mass *in vivo* (Ellingsgaard et al., 2008). The increase in IL-6 with each bout of exercise may be involved in protecting pancreatic β-cell mass and function.

## Other Muscle-Organ Cross-Talks

### Muscle-Brain Crosstalk

Regular exercise has beneficial effects on brain health (Cotman et al., 2007; Mattson, 2012). The fact that exercise is sensed by the brain suggests a direct crosstalk between working muscle and brain function (Pedersen and Febbraio, 2012; Benatti and Pedersen, 2015; Leardini-Tristao et al., 2019; Pedersen, 2019). Studies in humans (Erickson et al., 2011) and rodents (Kobilo et al., 2011) demonstrate a positive effect of exercise on hippocampus volume (Kobilo et al., 2011). In humans, brain-derived Neurotic factor (BDNF) Studies in humans demonstrate that BDNF is released from the brain in relation to exercise (Rasmussen et al., 2009; Seifert et al., 2010) and regular exercise for 3 months leads to an increase in the volume of hippocampus (Pajonk et al., 2010). In rodents, BDNF mRNA and protein increase in response to exercise (Pedersen and Febbraio, 2012; Benatti and Pedersen, 2015; Leardini-Tristao et al., 2019; Pedersen, 2019) and stimulate hippocampus growth (Loprinzi and Frith, 2019) as well as memory and learning (Vaynman et al., 2004a,b). Interesting studies in mice show that the myokines cathepsin-B (Moon et al., 2016) and irisin (Wrann et al., 2013) may be released from muscle to blood during exercise, passing from the blood to the brain and directly provoking an increase in brain BDNF. When IL-6 is centrally applied in mice, it suppresses feeding (Timper et al., 2017). Moreover, a much higher IL-6 concentration applied peripherally reduces the intake of food, suggesting that high systemic IL-6 concentrations may pass from the blood to the brain and regulate appetite. The latter results indicate that IL-6 released from muscle during exercise of high intensity and long duration (Febbraio and Pedersen, 2002), may lead to a decrease in appetite.

### Muscle-Muscle

Some myokines can exert their effects on the muscle itself. One of these is IL-6, which can work in both an endocrine and a paracrine manner within the muscle (Pedersen and Febbraio, 2008, 2012). In a metabolic perspective, studies in humans show that IL-6 is capable of increasing glucose uptake by a mechanism that involves activation of AMPK (Carey et al., 2006). Moreover, IL-6 increases insulin-stimulated glucose uptake *in vitro* as well as in *in vivo* in health humans (Carey et al., 2006). Furthermore, IL-6 increases fatty acid oxidation via AMPK activation (Kahn et al., 2005; Carey et al., 2006). BDNF is yet another myokine, which stimulates AMPK activation and thereby lipid oxidation. BDNF works in an autocrine or paracrine manner (Matthews et al., 2009). Finally, Musclin is an exercise-induced factor (Nishizawa et al., 2004) that promotes mitochondrial biogenesis in murine muscle (Subbotina et al., 2015).

### Muscle-Gut

IL-6 stimulates glucagon-like peptide-1 (GLP-1) secretion in mice from both pancreatic β-cells and intestinal L-cells, thereby enhancing insulin secretion. A recent human study from our group (Lang Lehrskov et al., 2018) demonstrates that IL-6 slows down the rate of gastric emptying. Thereby IL-6 indirectly exerts beneficial effects on postprandial glucose (Woerle et al., 2008).

### Muscle-Skin

Studies in exercising mice and humans suggest that muscle-derived IL-15 contributes to avoid aging of the skin (Crane et al., 2015). The latter study showed that that exercise regulates muscular IL-15 expression via skeletal muscle AMPK.

## The Potential Clinical Impact of Myokines in Immunometabolism

Myokines have been identified which include effects on e.g., lipid and glucose metabolism, browning of white fat, beta-cell-function, endothelial cell function and tumor growth. The biological and physiological identification of several myokines has identified these to be useful biomarkers for monitoring the exercise training, which is necessary in order to apply exercise as medicine for patients with specific diseases, such as diabetes, cardiovascular diseases and cancer. The identification of new myokines, playing specific roles in immunometabolism, may lead to new therapeutic targets for lifestyle-related diseases.

## Conclusion

During exercise, myokines play a role in regulating immune cell trafficking, inflammation and metabolism. Exercise training thereby represents a strategy to induce a anti-inflammation and improved metabolism, which may contribute to decrease the risk or progression of cancer and type 2 diabetes as well as other chronic disorders.

## Author Contributions

MB and BP wrote the manuscript. Both authors contributed to the article and approved the submitted version.

## Conflict of Interest

The authors declare that the research was conducted in the absence of any commercial or financial relationships that could be construed as a potential conflict of interest.
